# Estimation of and correction for finite motion sampling errors in small animal PET rigid motion correction

**DOI:** 10.1007/s11517-018-1899-8

**Published:** 2018-09-22

**Authors:** A. Miranda, S. Staelens, S. Stroobants, J. Verhaeghe

**Affiliations:** 1 Molecular Imaging Center Antwerp, University of Antwerp, Universiteitsplein 1, 2610 Antwerp, Belgium; 2 University Hospital Antwerp, Wilrijkstraat 10, 2650 Antwerp, Belgium

**Keywords:** Positron emission tomography, Motion correction, Image deconvolution

## Abstract

Motion tracking with finite time sampling causing an associated unknown residual motion between two motion measurements is one of the factors contributing to resolution loss in small animal PET motion correction. The aim of this work is (i) to provide a means to estimate the effect of the finite motion sampling on the spatial resolution of the motion correction reconstructions and (ii) to correct for this residual motion thereby minimizing resolution loss. We calculate a tailored spatially variant deconvolution kernel from the measured motion data which is then used to deconvolve the motion corrected image using a 3D Richardson-Lucy algorithm. A simulation experiment of numerical phantoms as well as a microDerenzo phantom experiment wherein the phantom was manually moved at different speeds was performed to assess the performance of our proposed method. In the motion corrected images of the microDerenzo phantom there was an average rod FWHM differences between the slow and fast motion cases of 9.7%. This difference was reduced to 5.8% after applying the residual motion deconvolution. In awake animal experiments, the proposed method can serve to mitigate the finite sampling factor degrading the spatial resolution as well as the resolution differences between fast-moving and slow-moving animals.

**Graphical abstract:**
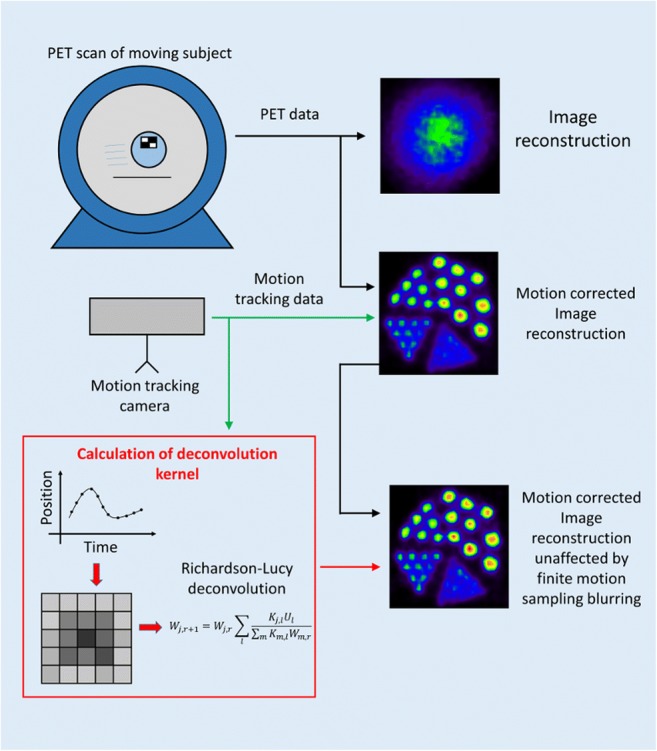
Motion correction of positron emission tomography (PET) scans of moving subjects can be performed by measuring the motion of the subject during the PET scan with an optical tracking camera. The motion tracking data obtained from the tracking camera is then used to correct the PET image reconstructions for motion. Due to finite time sampling of the motion data, the motion corrected reconstructions suffer from loss of spatial resolution. In the proposed method, a spatially variant deconvolution kernel is calculated from the motion tracking data, which is then used to correct the motion-corrected PET reconstructions for the blurring effect of the finite motion sampling through a Richardson-Lucy deconvolution.

Motion correction of positron emission tomography (PET) scans of moving subjects can be performed by measuring the motion of the subject during the PET scan with an optical tracking camera. The motion tracking data obtained from the tracking camera is then used to correct the PET image reconstructions for motion. Due to finite time sampling of the motion data, the motion corrected reconstructions suffer from loss of spatial resolution. In the proposed method, a spatially variant deconvolution kernel is calculated from the motion tracking data, which is then used to correct the motion-corrected PET reconstructions for the blurring effect of the finite motion sampling through a Richardson-Lucy deconvolution.

## Introduction

Small animal positron emission tomography (PET) is commonly used in preclinical in vivo research of neurological disorders such as Parkinson’s disease, Alzheimer’s disease, and Huntington’s disease [24] and psychiatric disorders such as depression, schizophrenia, and obsessive-compulsive disorder [17, 21]. During PET scanning the animals are generally immobilized using anesthesia to avoid image blurring that is associated with animal motion. However, the use of anesthesia can interfere with the neurological process under study and can thus affect the PET results [9]. To avoid this undesired and often unknown effect of anesthesia on the PET outcome, methods to perform PET studies of awake unrestrained animals are being developed [1, 22, 26].

Particularly for PET neuroimaging, rigid body motion correction can be used to perform scans of awake unrestrained animals. One of the methods to perform rigid body motion correction in PET image reconstruction consists of measuring the pose (position and orientation, 6 degrees of freedom) of the animal head during the PET scan with an external tracking device. Then, the motion information is used to spatially reposition all the lines of response (LORs) with respect to a single reference pose of the head [6, 15]. The repositioned LORs can then be rebinned (LOR rebinning) into sinograms or directly reconstructed with list-mode reconstruction [18].

Rigid motion correction has been successfully applied to awake rat brain PET studies by Kyme et al. [12] on a microPET Focus 220. However, the resolution in the final motion corrected image is still lower compared to a motion-free reference scan (see, e.g., Zhou et al. [28], Miranda et al. [16]). Among the reported factors that affect the quality and resolution of the motion corrected reconstructions are the following: the accuracy of the motion tracking device, the delay in the time synchronization between the motion measuring device and the PET scanner, the calibration error in the transformation between the tracking device and PET scanner coordinate system, the frame rate of the motion information (i.e., the sampling rate of the motion tracking), as well as the speed of the subject motion.

The loss of spatial resolution caused by the finite motion sampling arises from the fact that a single pose is assigned to all the LORs that fall within a single sampling interval, e.g., for a tracking frequency of 30 Hz all LORs within a time interval of 33 ms are repositioned using a single transformation from the measured pose to the reference pose. Thus, there exists a difference between the actual pose of the LOR and its assigned measured pose. The associated error depends on the speed of the subject motion and the tracking frame rate, with larger errors arising from faster motion and/or slower tracking frame rates. The resulting uncertainty in the positioning of the LORs causes a motion dependent loss of spatial resolution in the motion corrected reconstructions. In addition, the loss of resolution can differ throughout the motion corrected reconstruction due to spatially variant linear motion speed that is associated with rotational motion. Interpolation of the measured poses has been used previously to reduce the effect of the finite sampling interval. It was shown that this procedure increases the contrast in the motion corrected reconstructions [27].

The aim of this work is to provide a way to assess to what extent the finite time sampling of the motion tracking degrades the spatial resolution of the motion corrected reconstructions by estimating the residual motion kernel. Secondly, a correction method for the estimated resolution loss is proposed. Therefore, we develop a strategy to calculate the resolution kernel that models the resolution loss in image space that is caused by the LOR positioning uncertainty due to the finite sampling. The shape of the kernel will depend on the subject motion and tracking frame rate and can be calculated from the measured motion. This “residual motion” kernel can be calculated in a single voxel to provide a fast assessment of the resolution loss, e.g., in the center of the region of interest. The kernel can also be calculated for each individual image voxel. These kernels can then be used to perform “residual motion deconvolution” (RMD) after motion correction in order to correct for the resolution loss caused by the finite motion sampling. Finally, the motion corrected reconstructions deconvolved using this kernel will be compared with reconstructions using interpolated poses [23].

Image deconvolution methods have been previously used to perform motion correction in PET. In the method proposed by Faber et al. [4], the image motion blurring kernel of each voxel is calculated from the externally tracked motion data. The kernel thus models the blurring caused by the measured motion and the PET image is corrected using the Lucy-Richardson deconvolution using these kernels. Similarly, Rahmim et al. [19] use the same image motion blurring kernels and introduced them as part of the system matrix used in the maximum likelihood reconstruction. These methods are suitable when there is slight subject motion, as in the case of some human brain scans. However, for the case of small animal imaging, where the extent of the motion can be significantly higher, the use of motion blurring kernels would be impractical. Instead, here we performed first an event-by-event motion correction to compensate the measured component of the motion and then performed the residual motion deconvolution that models uncertainty in the measured motion due to the finite motion sampling.

## Methods

### PET scanning, motion tracking, LOR rebinning, and reconstruction

In this study, we used the Siemens Inveon microPET (Siemens Medical Solutions, Inc., Knokville, USA) scanner. The scanner consists of 80 rings (16 cm diameter) of 320 lutetium oxyorthosilicate (LSO) scintillation crystals with a size of 1.5 × 1.5 × 1.0-mm^3^ with a reconstructed image resolution of 1.5 mm [3, 25]. The scanner’s bore diameter is 12 cm.

**Fig. 1:**
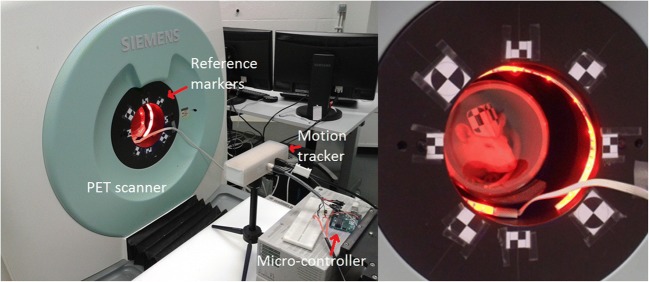
Setup for the awake rat scan, showing the MicronTracker, the Arduino microcontroller, the reference markers around the scanner bore, and the illumination for tracking of the subject (right panel)

For motion tracking during the PET imaging, a commercially available stereo optical tracking device (MicronTracker Sx60, Claron Technology Inc., Toronto, Canada) was used. The MicronTracker measures the pose of specially designed checkerboard markers in a reference frame relative to the camera position. Four markers of 2.4 × 2.4 mm^2^ and four of 1.2 × 1.2 mm^2^ were pasted on the scanner bore to be used as reference ([12]; Fig. 1). The motion of the subject marker was measured relative to these reference markers. The maximum tracking frame rate is 48 Hz, and the reported tracking accuracy by the manufacturer is 0.25 mm root mean square.

The spatial calibration between the MicronTracker and the PET scanner coordinate system is established by measuring the spatial location of a radioactive point source, attached to the center of a checkerboard marker in PET space, and the corresponding pose of the checkerboard marker in camera space for 20 different positions [13]. The transformation matrix between both coordinate systems is then determined using least squares regression. The same transformation matrix was used for all the experiments.

The time synchronization between the MicronTracker and the PET scanner is established using an Arduino Uno (Arduino SA) microcontroller to generate a regular square wave signal that triggers the MicronTracker, which in turn sends a signal to the microPET gating input. A few seconds before the end of the scan, the frequency of the gating signal changes with a predetermined pattern. This pattern is then used to relate each gate tag with its corresponding pose. The frequency of the square wave signal can be adjusted by the user to change the tracking frame rate. The tracking frame rate was set to 31.2 Hz in all experiments. Although a maximum tracking frame rate of 48 Hz is possible, 31.2 Hz was chosen due to the fact that at increased frame rate the camera exposure time reduces, increasing the noise in the images and therefore reducing the tracking accuracy [8].

**Fig. 2:**
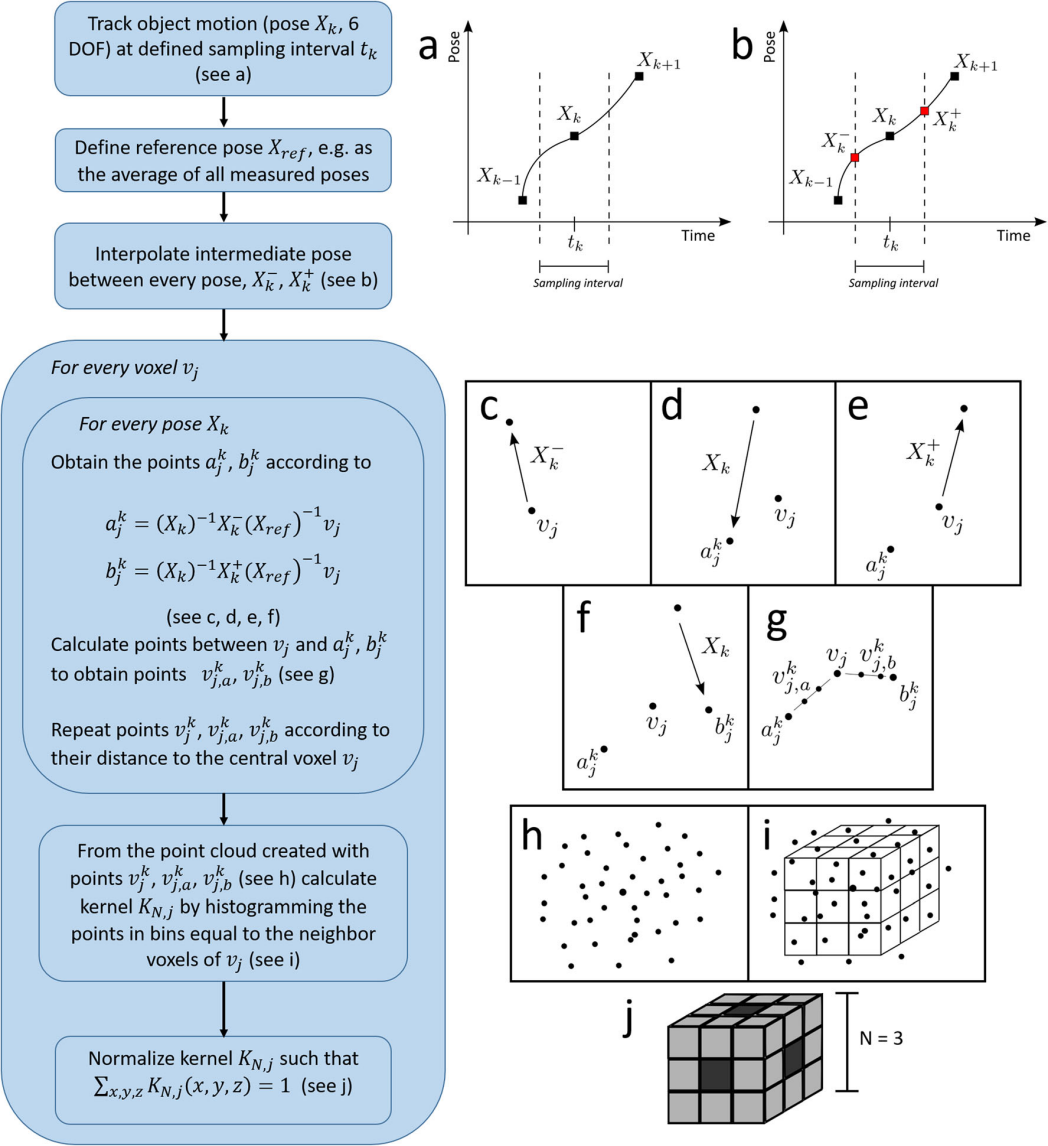
Algorithm diagram for the calculation of the residual motion kernels from the motion tracking data. Panels on the right side are explained in the algorithm diagram (left side). Symbols are defined in the text

For the motion corrected reconstructions, we have implemented the subsetized list-mode expectation maximization (OSEM) algorithm [18] with calculation of the sensitivity image by interpolation in the image space. The bore size of the Siemens Inveon microPET is substantially smaller than in previous implementations of awake small animal brain PET (12 cm compared to 22 cm for the microPET Focus 220 [12]). This poses challenges on the motion tracking; however, it does not impact the motion correction method as such. Briefly, in this method, each measured pose *X*_*k*_ (*k* = 1, …, *K* poses) is assigned to the group of LORs that are within the sampling interval centered around the time *t*_*k*_, the time that the pose was measured (Fig. 2a). These LORs, i.e., the lines connecting two coincidence events, are then moved to a reference pose *X*_ref_ by applying the transformation $M={X}_{\mathrm{ref}}{X}_k^{-1}$. The LORs in the reference pose are finally reconstructed using the OSEM algorithm without attenuation, scatters, or random correction. Resolution modeling was implemented in the image space [20] with a 1.2-mm Gaussian point spread function. These motion corrected images will then be deconvolved using our proposed residual motion kernel (see Section 2.2).

We compared our proposed deconvolution procedure to motion correction using pose interpolation of the motion tracking data [27]. The interpolated poses are calculated from the measured poses to assign a single position to each LOR (within the scanner time resolution of 0.2 ms), thus reducing the finite sampling interval. The interpolation of the pose position was obtained from cubic interpolation, while the pose orientation was calculated from cubic interpolation of the Euler angles [23].

### Residual motion kernel estimation and deconvolution

To calculate the residual motion kernel, the range of the residual motion that exists within each of the sampling intervals is estimated in image space. Each reference voxel location is transformed to the current measured position. The position uncertainty is then estimated by considering a position uncertainty distribution spanning from the voxel position that lie halfway between the measured voxel position in the current frame and previous frame and between the current and next frames respectively. To discretize the uncertainty distribution, a number of points are distributed over the uncertainty positions span. Finally, these points are transformed back to the reference orientation. Once this has been done for all frames, a kernel is fitted to the resulting point cloud. The detailed procedure is presented in the next paragraph.

For each measured pose *X*_*k*_ (*k* = 2, …, *K* − 1), the intermediate pose ${X}_k^{-}$ halfway between *X*_*k*_ and *X*_*k* − 1_, and the intermediate pose ${X}_k^{+}$ between *X*_*k*_ and *X*_*k* + 1_, are calculated (Fig. 2b). The location (*x*, *y*, *z*) of the intermediate pose is calculated using linear interpolation while for the orientation the mean rotation matrix is obtained through the singular value decomposition of the sum of the rotation matrices corresponding to the neighboring poses [7].

Then, to estimate the corresponding blurring in image space that the range of residual motion within the sampling interval causes, the intermediate poses ${X}_k^{-}$ and ${X}_k^{+}$ from the previous step are used to move the centers of the image voxels as follows:

1. All image voxels *v*_*j*_ (*j* = 1, …, *J*) are moved from their reference pose *X*_ref_, i.e., the pose of the motion corrected reconstruction (defined as the average from all measured poses), to the intermediate pose ${X}_k^{-}$. Then, all the image voxels are moved back towards the reference position according to the pose *X*_*k*_. In this way, the new voxel center coordinates ${a}_j^k$ are obtained, with slightly different position from the original coordinates due to the difference between the poses *X*_*k*_ and ${X}_k^{-}$, according to ${a}_j^k={\left({X}_k\right)}^{-1}{X}_k^{-}{\left({X}_{\mathrm{ref}}\right)}^{-1}{v}_j$.
2. Step 1 is repeated for all voxels using ${X}_k^{+}$ instead of ${X}_k^{-}$ obtaining voxel center coordinates ${b}_j^k$, i.e., ${b}_j^k={\left({X}_k\right)}^{-1}{X}_k^{+}{\left({X}_{\mathrm{ref}}\right)}^{-1}{v}_j$.
3. The line connecting the points *v*_*j*_ and ${a}_j^k$ is then calculated. At intervals with length *d*_*m*_ (average of the voxel length in the *x*, *y* and *z* direction) from the point *v*_*j*_, a point is calculated obtaining the points ${v}_{j,a1}^k$, ${v}_{j,a2}^k$, …, with the number of point dependent on the length of the line. Likewise for the line connecting points *v*_*j*_ and ${b}_j^k$, at intervals with length *d*_*m*_ from the point *v*_*j*_ a point is calculated obtaining the points ${v}_{j,b1}^k$ and ${v}_{j,b2}^k$.
4. Depending on the predefined size *N* of the deconvolution kernel that is calculated, the central voxel ${v}_j^k$ is repeated ⌈*N*/2⌉ times, voxels ${v}_{j,a1}^k$ and ${v}_{j,b1}^k$ ⌈*N*/2⌉ − 1 times and so on. This procedure is made in order to assign more weight to the points closer to the voxel center.
5. Steps 1 to 4 are repeated for all the poses *k* = 2, …, *K* − 1, and for each voxel *v*_*j*_, we obtain an associated point cloud with the coordinates ${v}_j^k$, ${v}_{j,a}^k$, and ${v}_{j,b}^k$, with each point repeated the corresponding number of times (step 4).
6. The deconvolution kernel *K*_*N*, *j*_ for each voxel *v*_*j*_ of size *N* is calculated from the point cloud ${v}_j^k$, ${v}_{j,a}^k$ and ${v}_{j,b}^k$ (*k* = 2, …, *K* − 1) by computing the 3D histogram of the points with bins equal to the neighbor voxels of ${v}_j^k$.
7. The 3D residual motion kernel *K*_*N*, *j*_ is finally normalized so that ∑_*x*, *y*, *z*_*K*_*N*, *j*_(*x*, *y*, *z*) = 1.

The use of points to represent the motion path of the voxel due to the residual motion was preferred over a ray intersection with the image voxels to reduce calculation time.

The deconvolution kernel *K*_*N*, *j*_ of size *N* is truncated and normalized if the residual motion is wider. Therefore, to evaluate the impact of the kernel size, three types of kernels, with kernel size *N* = 3, 5, and 7, were calculated. These kernels will be referred to as *K*_3_, *K*_5_, and *K*_7_, respectively.

The kernel can be calculated in a single voxel of interest for a fast assessment of the resolution loss in a particular position. If the kernels are however calculated for each voxel in the region of interest, they can be used in a 3D Richardson-Lucy (RL) deconvolution algorithm [14] to deconvolve the motion corrected reconstructions:

$$ {W}_{j,r+1}={W}_{j,r}\sum \limits_l\frac{K_{j,l}{U}_l}{\sum_m{K}_{m,l}{W}_{m,r}} $$

where *W*_*j*, *r*_ is the image value of the *j*th voxel in the deconvolved image at the *r*th iteration, *K*_*j*, *l*_ is the contribution of the kernel in the *j*th position to the *l*th voxel, and *U*_*l*_ is the *l*th voxel value of the motion corrected reconstruction before deconvolution. In the RL deconvolution, a Poisson noise model is assumed. We will refer to the deconvolution with the residual motion kernel as residual motion deconvolution (RMD).

In our experiments, the kernel calculation and deconvolution were performed only on the region of interest that contained the object. The kernel calculation and the deconvolution were implemented in MATLAB Release 2012b (The Mathworks, Inc., Natick, USA).

#### Spatially variant resolution and residual motion kernel shape validation

To illustrate the spatially variant resolution loss after motion correction and the corresponding spatially variant residual kernel, a 48.9-mm long rod (1 voxel wide) with uniform activity was simulated. The rod was placed along the *y* axis with one of its end points in the center of the coordinate system (i.e., center of the scanner field of view, CFOV). The rod was then rotated about the *x* axis considering a sinusoidal signal with frequency of 0.5 Hz and amplitude of 25.5°. Back-to-back photons escaping from the moving phantom were simulated. For this simulation, the motion was sampled with a timing resolution of 1 ms. The simulated data was then motion corrected with a motion sampling interval of 32 ms. The FWHM of the static and the motion corrected reconstructions was measured along the *z* axis, and its difference was calculated in function of the distance along the *y* axis. In addition, the kernel *K*_5_ was calculated at several voxel positions along the rod and the kernel FWHM along the *z* axis was measured from a Gaussian fit to the kernel profile.

### Numerical mouse brain phantom simulation

In order to asses to which degree the residual motion blurring affects the regional brain quantification in two animal conditions with different levels of locomotion, a numerical mouse brain phantom experiment was performed. Motion data from a head motion tracking experiment in naïve and memantine treated mice placed inside the PET scanner was used to calculate the residual motion kernels. Memantine administration has been shown to significantly increase mouse locomotion in comparison with a naïve condition [5]. Then, to estimate the effect of the residual motion in the two conditions on brain quantification, the kernels were used to blur a mouse brain numerical phantom. The numerical mouse brain phantom was based on a template in Waxholm space [11]. Increased striatal uptake was simulated with a 1.65 to 1 ratio (striatum versus rest of the brain). To quantify the effect, average regional striatal uptake after blurring with the residual motion kernel was calculated in the reference image as well as in both animal conditions blurred images.

### Simulation experiment

A simulated dataset was generated by combining list mode data of a static (i.e., motion-free) PET scan of a microDerenzo phantom and previously measured motion data of a manually moved microDerenzo phantom. The LORs in the list mode data were then moved according to this motion to generate simulated PET data that was affected by subject motion. The microDerenzo phantom (diameter 30 mm, height 13 mm) had six rod sections with an inner diameter of 1.25, 1.5, 2, 2.5, and 3 mm respectively. The scan time was 10 min, and the activity was 12.9 MBq of [^18^F]-FDG. The motion data was previously measured using the MicronTracker in a phantom experiment where the phantom was moved manually. The motion was sampled every 32 ms, and the average measured motion speed was 74 mm/s. The list mode data has a temporal resolution of 0.2 ms, and to simulate continuous motion, the original motion was interpolated (using cubic interpolation) to obtain 1 ms sampling intervals. The list mode data of the motion-free scan was moved according to the interpolated motion, i.e., with 1 ms sampling intervals. Then, these displaced LORs were corrected for motion according to the original measured motion, i.e., with bin intervals of 32 ms. Thus, all the LORs in the 32 ms interval that have been moved with different poses (every 1 ms) are being corrected using a single pose corresponding to the central pose of the interval. A second simulated dataset was generated by considering sampling intervals of 64 ms for the motion correction. This would equally correspond to the situation where the motion speed doubles.

The errors present in this motion corrected simulation is caused uniquely by the difference between the actual pose of the LOR and the central pose of the corresponding interval that is used for the motion correction. All the other factors that can affect the spatial resolution in the motion correction technique (e.g., calibration error, tracking accuracy, synchronization delay) are not simulated.

To assess the different kernel sizes, the kernels *K*_*N*_ (*N* = 3, 5, 7) were calculated for the 32 ms sampling interval data. The different kernels were then used in the residual motion deconvolution of the motion corrected reconstruction. The average FWHM, taken in tangential direction, and the average peak-to-valley ratio (PVR) of all the rods of 2, 2.5, and 3 mm were measured in the deconvolved images for eight iterations of the residual motion deconvolution. The FWHM was calculated from a Gaussian fit to the profiles through the individual rods of the same size and was then averaged to obtain the average FWHM. The FWHM and PVR values were then compared to the average FWHM and PVR of the rods in the reference reconstruction of the motion-free scan. The data for the 64 ms sampling interval simulation was analyzed similarly as aforementioned.

### MicroDerenzo phantom experiment

To further evaluate the performance of the deconvolution on real measured data, two microDerenzo phantom experiments whereby the phantom was moved manually at two different speeds were considered. The same phantom as described in Section 2.3 was used. The phantom was filled with 12.9 MBq of [^18^F]-FDG, and scan duration was 10 min. During the first scan, the phantom was moved more slowly while during the second scan, the phantom was moved with a faster speed. The motion was measured with the MicronTracker device at a frame rate of 31.2 Hz (32 ms bins) with a checkerboard marker of 36 × 30 mm^2^. At the end of the two motion experiments, a third reference static scan, i.e., without any motion, was made for evaluation purposes.

The reconstructions after motion correction were deconvolved for residual motion using kernel *K*_5_ with eight RMD iterations. The average FWHM and PVR values for the 2, 2.5, and 3 mm rods were determined and compared to the values of the motion-free scan.

In addition, a motion correction of the slow and fast motion scan was performed using interpolated poses as detailed in Section 2.1.

The region of interest where the deconvolution was performed contained 45 × 43 × 24 voxels, resulting in 46,440 kernels.

### Resolution loss quantification

We quantify the loss of spatial resolution due to the residual motion blurring through the FWHM and PVR of the microDerenzo phantom rods, with the values of the motion-free case as the reference values.

In addition, to quantify the loss of spatial resolution of the motion corrected reconstructions in comparison with the motion-free reconstructions, a scale parameter [2] was calculated as follows. A mask covering the region where the loss of spatial resolution is to be assessed is defined by using an activity threshold in both motion corrected and motion-free images. Then, the motion-free image is filtered with a spatially invariant Gaussian filter with a scale parameter (*σ*^2^) ranging from 0 to 2.88 mm^2^. The image correlation between the filtered motion-free and motion corrected images is calculated. Finally, the scale parameter value of the filtered motion-free image with maximum correlation with the motion corrected image is selected as the corresponding scale parameter for that motion corrected image. Although the loss of spatial resolution can vary over the image, the scale parameter serves as a metric of the average loss of spatial resolution.

To investigate the shape of the estimated deconvolution kernel *K*_5_, the principal axes of the average kernel were calculated for all experiments as follows. The deconvolution kernels for all voxels in the region of interest of each experiment were summed. Then, the central image moments [10] of the summed kernels ${\overline{K}}_5$ were calculated to create the image covariance matrix:

$$ \mathit{\operatorname{cov}}\left[{\overline{K}}_5\right]=\left[\begin{array}{ccc}{\mu}_{2,0,0}^{\hbox{'}}& {\mu}_{1,1,0}^{\hbox{'}}& {\mu}_{1,0,1}^{\hbox{'}}\\ {}{\mu}_{1,1,0}^{\hbox{'}}& {\mu}_{0,2,0}^{\hbox{'}}& {\mu}_{0,1,1}^{\hbox{'}}\\ {}{\mu}_{1,0,1}^{\hbox{'}}& {\mu}_{0,1,1}^{\hbox{'}}& {\mu}_{0,0,2}^{\hbox{'}}\end{array}\right] $$

where *μ*^′^ is the image central moment where the subindex indicates the order of the moment in the *x*, *y*, and *z* direction respectively. Finally, from the covariance matrix, the eigenvalues and eigenvectors were calculated to obtain the magnitude (standard deviation) and direction of the kernel principal axes.

## Results

### Spatially variant resolution and residual motion kernel shape validation

**Fig. 3:**
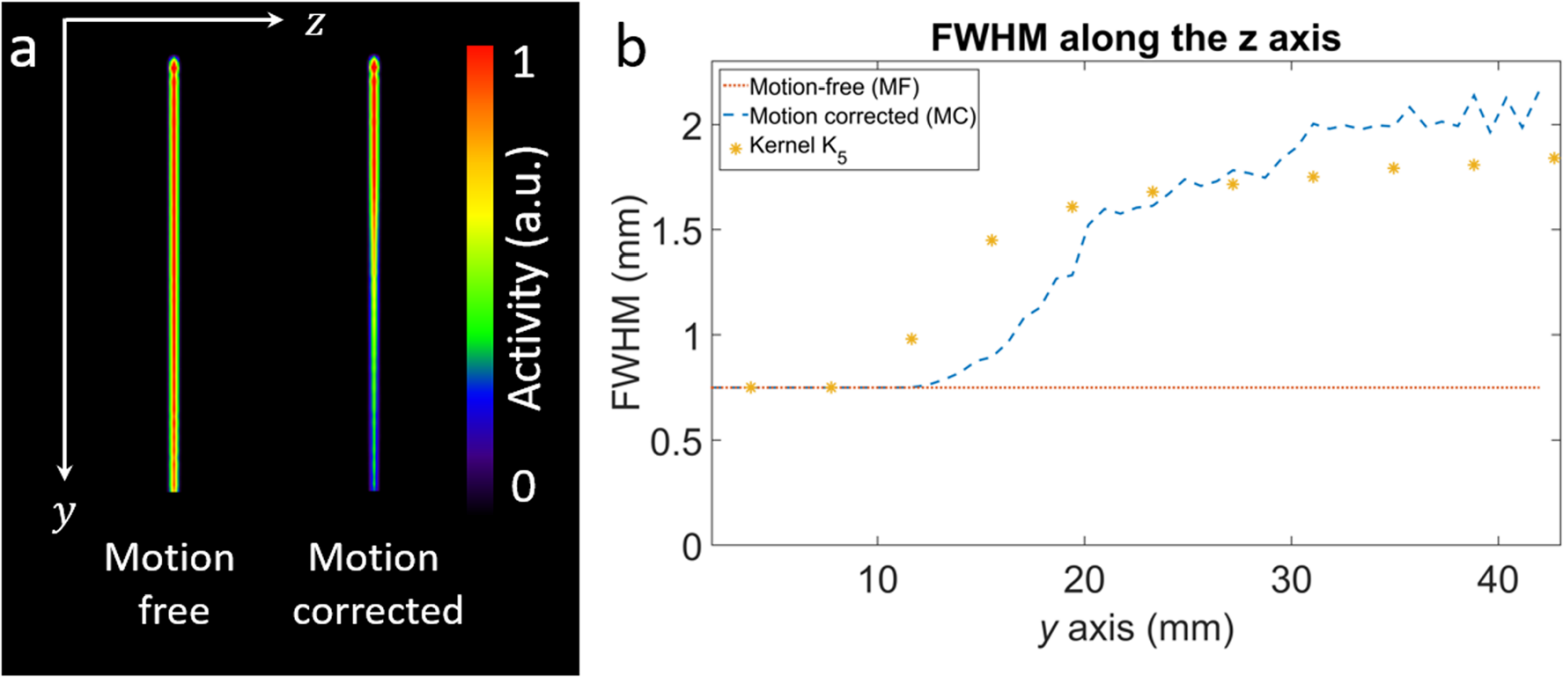
**a** Reconstruction of the simulated motion-free and motion corrected rod phantom. **b** FWHM along the *z* axis of the motion-free and motion corrected rod phantom reconstructions and the FWHM of the estimated kernel *K*_5_

The reconstruction of the motion-free and motion corrected rod together with the FWHM measured along the *z* axis is shown in Fig. 3. The measured FWHM in the motion corrected reconstruction increases as a function of the distance from the origin. At a distance of 3.11, 21.0, and 41.1 mm from the origin, the FWHM error with respect to the motion-free scan is 0, 0.84, and 1.24 mm respectively. The FWHM of the estimated deconvolution kernel *K*_5_ follows the measured FWHM of the motion corrected reconstruction. At the origin of the coordinate system, the shape of the deconvolution kernel is an impulse, as there is no residual motion to correct for in that location (zero tangential speed). As we move away from the origin along the *y* axis direction, the tangential speed in the *z* axis direction increases and the shape of the kernels becomes wider in that direction. Along the *x* and *y* directions, the shape of the kernel is an impulse, as the tangential speed is approximately zero in the *y* direction and equals zero in the *x* direction.

### Numerical mouse brain phantom simulation

**Fig. 4:**
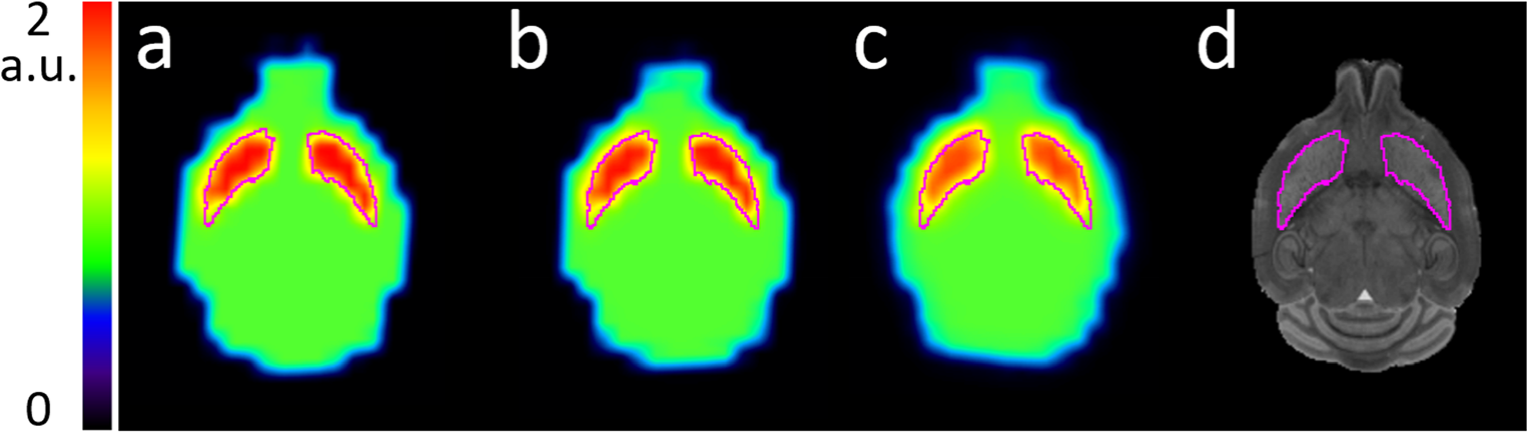
Transverse slices of the numerical mouse brain phantom with activity in the striatum, delineated in purple. Numerical phantom (**a**) reference (unblurred), (**b**) naïve mouse motion blurred, (**c**) memantine challenge motion blurred, and (**d**) MRI for anatomical reference

The measured average speed of the mouse in the naïve condition was 1.55 cm/s, while for the memantine challenge condition, it was 4.57 cm/s. Figure 4 shows the numerical mouse brain phantom images after blurring by the residual motion kernels in all conditions.

The average striatum activity in the reference image is 1.65. After residual motion blurring, the average striatum activity reduces to 1.64 and 1.52 for the naïve and memantine challenge conditions respectively, i.e., a difference of − 0.61% and − 7.9% with respect to the reference. The difference between naïve and challenge conditions is 7.3%.

### Simulation experiment

**Fig. 5:**
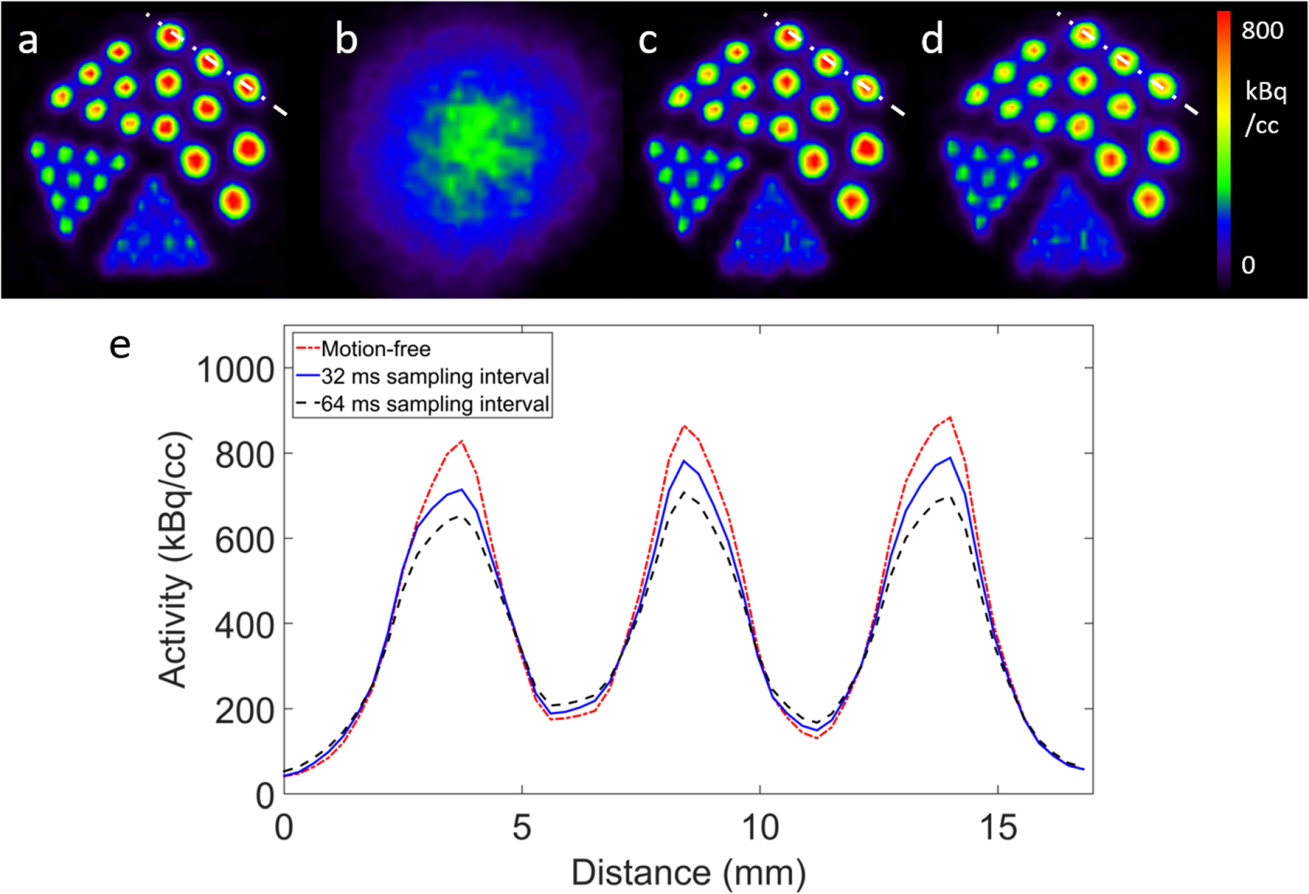
(**a**) Reconstruction of the motion-free scan. (**b**) The uncorrected motion scan. Motion corrected reconstructions for the simulations with a motion sampling interval of (**c**) 32 ms and (**d**) 64 ms. (**e**) Profiles through the 2.5 mm rods taken through the white dotted lines

In Fig. 5, the reconstruction of the reference motion-free scan is shown together with the reconstructions of the uncorrected simulated data and the reconstruction after motion correction using both motion sampling intervals. The motion corrected reconstructions show degradation of the spatial resolution and contrast compared to the motion-free reconstruction. As can be seen from the profiles, there is a higher degradation for the 64 ms sampling interval case compared to the 32 ms case, as was expected. The average peak-to-valley ratio of the 2.5 mm diameter rods in the motion-free scan is 5.75, in comparison with 4.61 and 3.74 for the corrected reconstructions with 32 and 64 ms sampling intervals respectively. As the simulation with the 64 ms bin intervals also corresponds to a sampling at 32 ms but with the motion speed doubled, this shows that the performance of the motion correction without additional residual motion deconvolution is motion dependent.

**Fig. 6:**
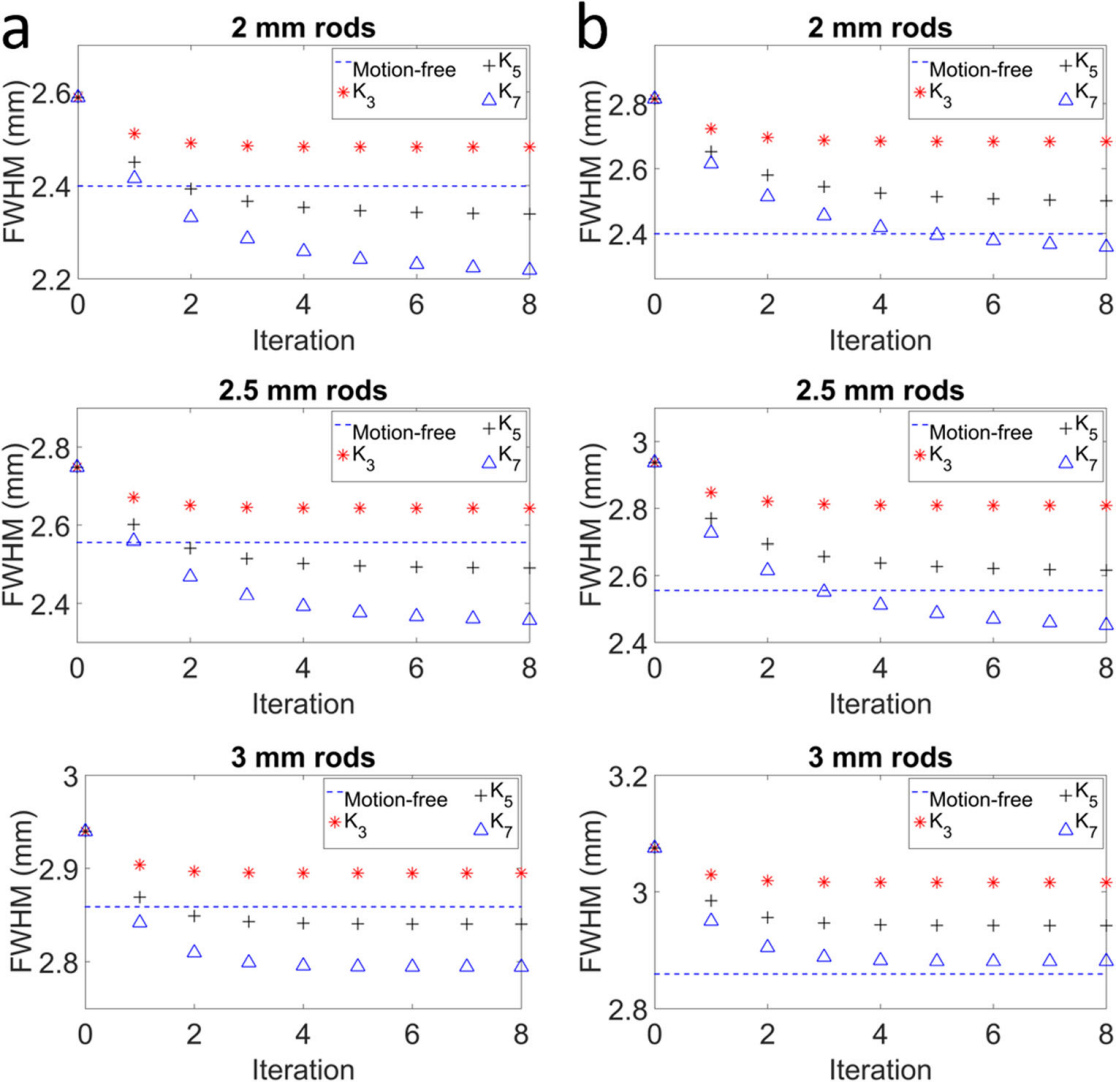
Average FWHM for the 2, 2.5, and 3 mm rod sizes after eight iterations of the RMD algorithm using the different deconvolution kernels in the motion correction simulation. Column **a** for sampling intervals of 32 ms and **b** for 64 ms.

Figure 6a shows the FWHM measured for the 2, 2.5, and 3 mm rod sizes for the case of the 32 ms sampling interval as a function of the RMD iteration number. For all kernels, the FWHM converges after eight iterations for all three rod sizes. Depending on the kernel size, the deconvolution overcompensates or undercompensates the resolution loss. After eight iterations, for the 2.5 mm rods, the difference with the motion-free reference scan FWHM is 3.53%, 2.35%, and 7.84% for kernels *K*_3_, *K*_5_, and *K*_7_ respectively. For the 2, 2.5, and 3 mm rods, kernel *K*_5_ results in a FWHM that most closely resembles the FWHM in the reference scan with a 2.5%, 2.35%, and 0.66% difference respectively. For all rod sizes, *K*_7_ results in the most severe overestimation.

Figure 6b shows the comparison of the average FWHM of the rods after RMD with kernels *K*_3_, *K*_5_, and *K*_7_ for the 64 ms sampling intervals. Kernel *K*_7_ results in the FWHM value that is closest to the FWHM of the motion-free reconstruction for the 2 and 3 mm rod sizes. For the 2.5 mm rods, the optimal kernel is *K*_5_. The difference with the motion-free reference scan was 1.7%, 2.39%, and 0.77% for the 2, 2.5, and 3 mm rod sizes using the optimal kernel in each case. For all three rod sizes, kernel *K*_3_ underestimates the FWHM by more than 5% for the 64 ms sampling interval case.

As was the case for the FWHM, the PVR values converged after eight iterations. Table 1 summarizes the average PVR for the 2, 2.5, and 3 mm rod sizes for the motion correction simulation deconvolutions with sampling intervals of 32 ms at iteration 8. For the 2 and 2.5 mm rods, kernel *K*_3_ results in the PVR that was closest to the value in the motion-free case, while for the 3 mm rods, kernels *K*_5_ result in a PVR that is closest to the reference motion-free value.

**Table 1:** Average peak-to-valley ratio (± 1 standard deviation) of the 2, 2.5, and 3 mm rods of the motion correction simulation with sampling interval of 32 ms at iteration 8

Rod radius (mm)	2	2.5	3
Motion-free	3.76 ± 0.57	6.31 ± 0.19	8.51 ± 0.47
*K*_3_	3.27 ± 0.55	5.72 ± 0.24	7.83 ± 0.37
*K*_5_	4.68 ± 0.79	7.10 ± 0.34	8.62 ± 0.42
*K*_7_	5.83 ± 1.00	9.07 ± 0.47	9.43 ± 0.47

In the 64 ms case, RMD using kernel *K*_5_ results in the PVR value that most closely resembles the PVR of the motion-free reference reconstruction for the 2 and 2.5 mm rod sizes and kernel *K*_7_ for the 3 mm rods. The highest difference occurs in the 2.5 mm rods (11.9%) and the lowest in the 2 mm rods (1.3%).

Although the optimal kernel to correct for the residual motion loss of spatial resolution depends on the structure size (rod diameter) and the sampling interval, for the sampling interval used in the following experiments (32 ms), RMD using the kernel *K*_5_ results in reconstructions that most closely resemble the motion-free reconstruction. For this reason we have selected this kernel for the phantom and the in vivo experiments.

### MicroDerenzo phantom experiment

**Fig. 7:**
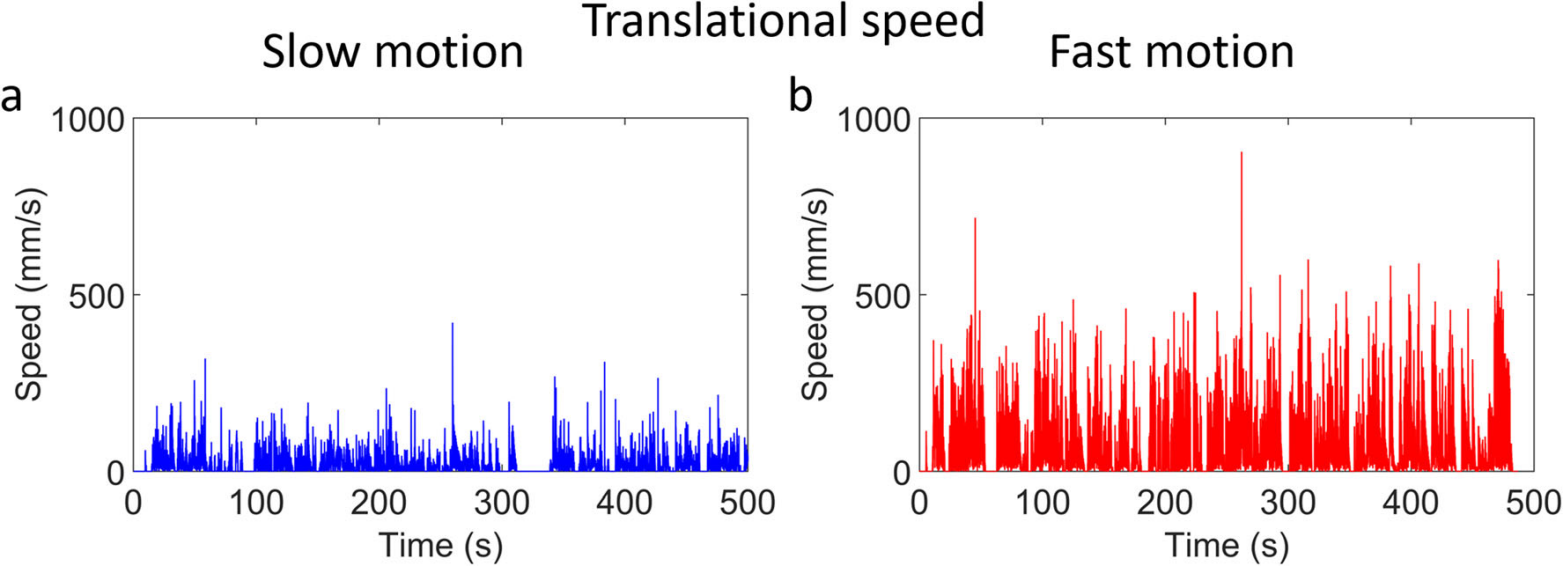
Translational speed of the **a** slow and **b** fast motion microDerenzo phantom scans

The translational speed (including displacement caused by rotational motion) at the center of the phantom throughout the scan of the microDerenzo phantom is shown in Fig. 7 for the slow and fast motion cases respectively. For the slow motion scan, the average displacement speed was 20 mm/s (0.66 mm per 32 ms bin) with a maximum of 420 mm/s. For the fast motion scan, the average displacement speed was 74 mm/s (2.44 mm per 32 ms bin) with a maximum of 904 mm/s.

For a volume of 45 × 43 × 24 voxels, the calculation of the kernel took approximately 20 min on an Intel Core i7 2.5 GHz processor. Eight iterations of the RMD took approximately 1 min using the 5 × 5 × 5 voxel kernel.

**Fig. 8:**
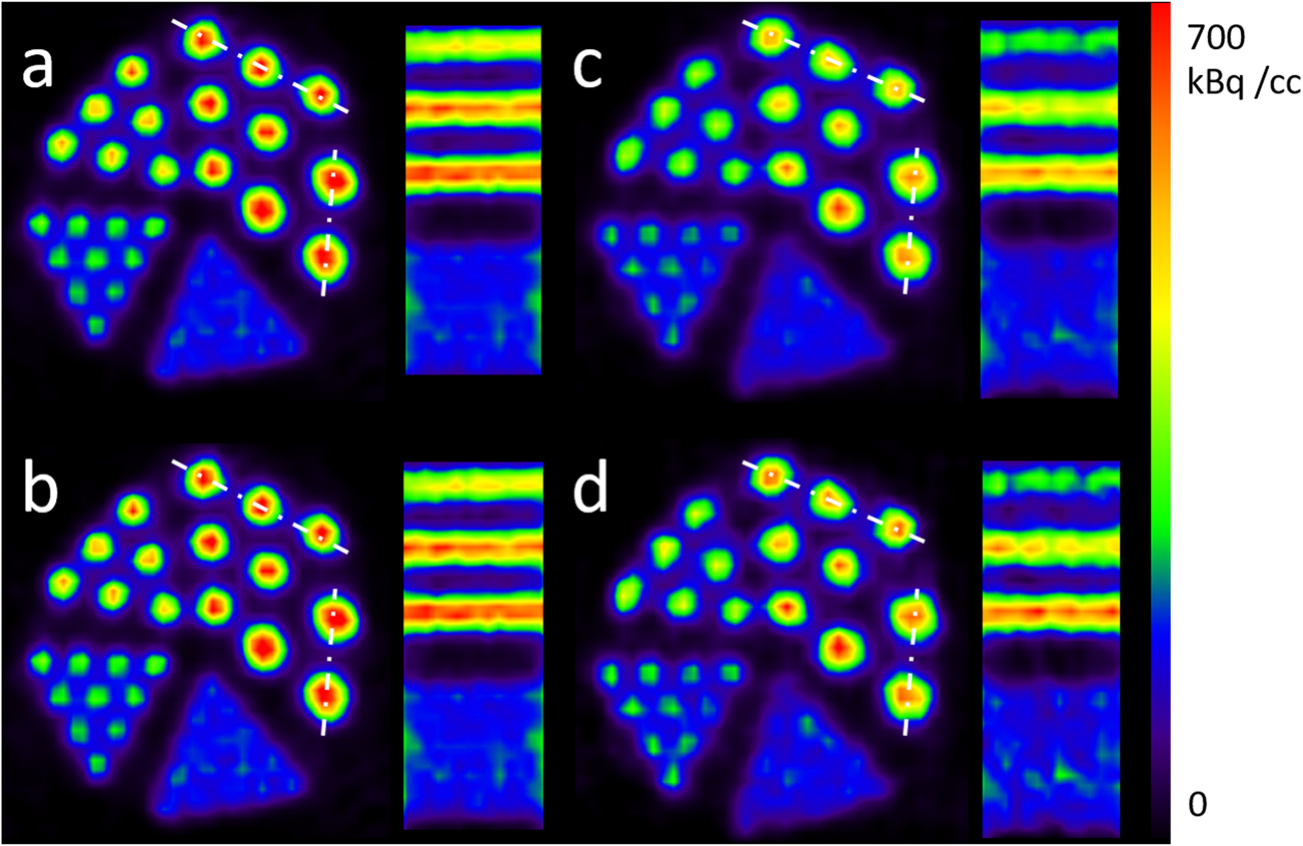
Transverse and coronal slices through the motion corrected reconstruction of the microDerenzo phantom scan for the slow motion case (**a**) without and (**b**) with RMD, and motion corrected reconstruction of the fast motion case (**c**) without and (**d**) with RMD.

**Fig. 9:**
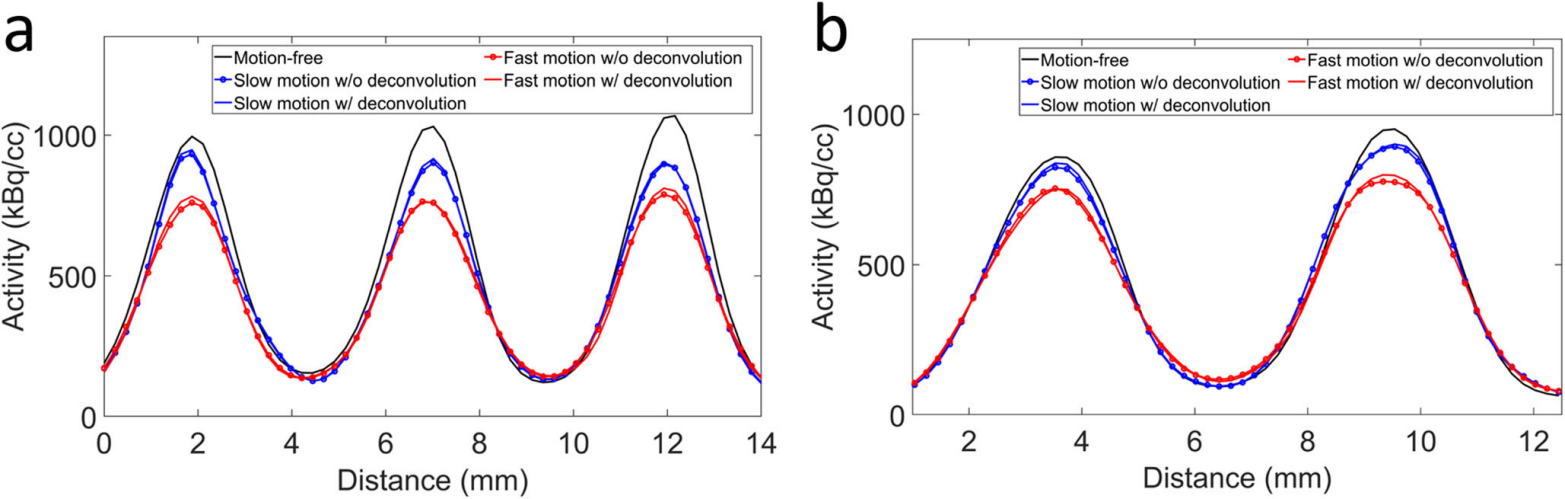
Profiles through the (**a**) 2.5 and (**b**) 3 mm rods of the microDerenzo phantom for the motion-free, slow and fast motion case after motion correction, without and with RMD

In Fig. 8, the reconstructions of the slow and fast motion scans after motion correction and after eight iterations of the RMD with kernel *K*_5_ are shown. The profiles through the 2.5 and 3 mm rods along the white lines in Fig. 8 are shown in Fig. 9. It can be seen that the loss of spatial resolution and contrast is more severe for the case of the fast motion scan in comparison with the slow motion scan.

The average FWHM of the 2, 2.5, and 3 mm rods for the different reconstructions is shown in Table 2. Compared to the motion-free reconstruction, the FWHM of the motion corrected reconstruction of the slow motion case is increased by 6.25% and 3.14% for the 2 and 2.5 mm rods respectively. For the 3 mm rods, there is no increase in FWHM. After RMD, the FWHM was increased with respect to the motion-free case by only 4.60% and 1.57% for the 2 and 2.5 mm rods respectively. For the fast motion case, the FWHM of the 2, 2.5, and 3 mm rods is increased by 18.7%, 15.3%, and 6.29% respectively for the motion corrected compared to the motion-free reconstruction. After RMD, the FWHM was increased with respect to the motion-free case by only 12.1%, 8.24%, and 2.45%. The difference between the FWHM of the fast and slow motion cases of the motion corrected reconstructions before RMD is 11.1%, 11.1%, and 6.8% for the 2, 2.5, and 3 mm rods respectively. After RMD, these differences are reduced to 6.15%, 6.36%, and 4.18% respectively.

**Table 2:** Average FWHM (mm, ± 1 standard deviation) of the 2, 2.5, and 3 mm rods of the motion-free recontruction and of the motion corrected images for the slow and fast motion cases without and with (w/) RMD. The results for the motion correction using interpolated poses are also shown

Rod size	2		2.5		3	
Motion-free	2.40 ± 0.18		2.55 ± 0.08		2.86 ± 0.10	
	Slow motion	Fast motion	Slow motion	Fast motion	Slow motion	Fast motion
MC	2.55 ± 0.05	2.85 ± 0.10	2.63 ± 0.08	2.94 ± 0.11	2.84 ± 0.07	3.04 ± 0.03
MC w/ RMD	2.51 ± 0.05	2.69 ± 0.07	2.59 ± 0.07	2.76 ± 0.09	2.81 ± 0.07	2.93 ± 0.03
MC w/ interp. poses	2.52 ± 0.06	2.68 ± 0.09	2.60 ± 0.08	2.78 ± 0.06	2.82 ± 0.08	2.95 ± 0.07

Finally, the FWHM values for the three rod sizes of the motion corrected reconstructions using interpolated poses were similar (within the standard deviation) to the rods FWHM obtained using RMD.

The average PVR for the 2, 2.5, and 3 mm rods is summarized in Table 3. As could be expected, the PVR values after motion correction for the fast motion case are lower compared to the slow motion case. However, after deconvolution, the PVR values are closer to the PVR values of the motion-free reconstruction. In addition, the PVR values for the slow and fast motion are closer to each other after the deconvolution. Similar to what was found using the FWHM metric, the PVR of the motion corrected reconstructions using interpolated poses was similar (within the standard deviation) to the PVR obtained using RMD.

**Table 3:** Average peak-to-valley ratio (± 1 standard deviation) of the 2, 2.5, and 3 mm rods of the motion-free and the motion corrected reconstructions for the slow and fast motion case without and with (w/) RMD. The results for the motion correction using interpolated poses is also shown

Rod size	2		2.5		3	
Motion-free	3.76 ± 0.57		6.31 ± 0.19		8.51 ± 0.47	
	Slow motion	Fast motion	Slow motion	Fast motion	Slow motion	Fast motion
MC	3.17 ± 0.32	2.10 ± 0.23	5.37 ± 0.13	3.59 ± 0.22	7.67 ± 0.41	5.60 ± 0.22
MC w/ RMD	3.29 ± 0.33	2.38 ± 0.28	5.68 ± 0.15	4.42 ± 0.28	8.01 ± 0.43	6.33 ± 0.27
MC w/ interp. poses	3.28 ± 0.34	2.41 ± 0.30	5.60 ± 0.15	4.41 ± 0.29	7.85 ± 0.47	6.08 ± 0.30

### Resolution loss quantification

Table 4 shows the scale parameter and magnitude and direction of the average kernel ${\overline{K}}_5$ principal axes for all experiments. For the microDerenzo phantom experiments, the loss of spatial resolution is greater for the 64 ms motion sampling and the fast motion cases in comparison with 32 ms sampling and slow motion case respectively. For the real data experiments, factors other than the finite motion sampling error further degrade the spatial resolution.

**Table 4:** Scale parameter (mm^2^) and principal axis magnitude (mm) and corresponding direction of the average RMD kernel ${\overline{K}}_5$ for all experiments

Experiment	Scale parameter (*σ*^2^)	Principal axis magnitude	Principal axis direction
Rod simulation	0.146	0, 0.040, 0.611	(1, 0, 0), (0, − 1, 0), (0, 0, 1)
microDerenzo simulation 32 ms	0.146	0.338, 0.466, 0.481	(− 0.151, − 0.975, 0.165), (0.963, − 0.107, 0.246), (0.222, − 0.196, − 0.955)
microDerenzo simulation 64 ms	0.199	0.374, 0.520, 0.531	(− 0.152, − 0.976, 0.152), (0.661, 0.014, 0.750), (− 0.735, 0.215, 0.643)
microDerenzo slow motion	0.260	0.149, 0.180, 0.338	(0.254, 0.953, − 0.162), (− 0.939, 0.283, 0.194), (0.231, 0.103, 0.967)
microDerenzo fast motion	0.379	0.308, 0.375, 0.531	(0.144, − 0.988, 0.037), (0.963, 0.132, − 0.235), (0.228, 0.069, 0.971)

For the kernel principal axes magnitude, the value is greater for the 64 ms motion sampling and fast motion cases in comparison with the 32 ms and slow motion cases respectively.

The shape of the kernel is highly asymmetric for the rod simulation phantom experiment, where the magnitude of the principal axis is a lot higher in the *z* direction, the direction in which the applied motion was maximum, in comparison with the other two axes. For all other experiments, some asymmetry is present but in a lower degree.

## Discussion

In PET rigid motion correction, the image resolution depends on the frame rate of the tracking device and on the subject motion speed. This effect was quantified and demonstrated in simulation and phantom experiments. In a simulation of a numerical mouse brain phantom, using real mouse motion data, activity in the striatum was 7.3% lower in the memantine challenge (more and faster head motion during scanning) than in the naïve condition. This difference was only attributed to the residual motion effect. Therefore, this would represent a spurious change in the underlying biological response in striatal uptake between both animal conditions. By correcting for the residual motion blurring, the spurious change would not be observed.

In simulation experiments, the motion corrected reconstructions with a motion sampling interval of 64 ms shows greater loss of spatial resolution compared to the motion corrected reconstructions with a motion sampling interval of 32 ms as could be anticipated. The slower sampling rate is equivalent to a faster motion speed of the subject. Similarly, in the phantom experiments, the motion corrected reconstructions for the fast motion case show increased resolution loss compared to the slow motion case. These results are in agreement with findings in Kyme et al. [12]. The scan-dependent image resolution in the motion corrected images is an undesirable effect. To counteract this effect, we have proposed to apply an additional deconvolution after image reconstruction, the residual motion deconvolution, using a motion dependent and spatially variant resolution kernel. The kernel models the residual motion that is present between the samples of the motion measurements. Our simulation and phantom experiments have shown that the RMD successfully compensates for some of the resolution loss due to the finite sampling error of the motion measurements and, importantly, the final resolution as measured by the FWHM and PVR is less dependent on the tracking frame rate or motion speed after applying the RMD.

In the motion simulation experiments, for the 32 ms sampling interval, the optimal kernel was determined to be *K*_5_ (5 × 5 × 5 voxels) through the measurement of the FWHM and PVR of a resolution phantom. The level of residual motion compensation can be inferred by the shape and size of the RMD kernel. Wider kernels result in increased compensation than narrower kernels. The 3 × 3 × 3 kernel *K*_3_ is too small to capture the wide residual motion blurring in both the 32 and 64 ms sampling cases. For the 32 ms case, the 7 × 7 × 7 kernel *K*_7_ resulted in overcompensation of the spatial resolution loss compared to kernel *K*_5_. When the motion that is needed to compensate for is present in a smaller region than the 5 × 5 × 5 voxel neighborhood, the 7 × 7 × 7 voxel kernel is not necessary. However, when the motion to compensate for is present in a wider region, the 5 voxel kernels will truncate the motion compensation and it will undercompensate the motion. For these cases, the 7 voxel kernels are more suitable. For the 64 ms case, for the 2 and 3 mm rods, kernel *K*_7_ resulted in the closest FWHM to the motion-free case.

Another aspect that is of critical importance in an iterative deconvolution strategy is the iteration number. In our simulations, for all analyzed rod sizes and for the two simulated motion sampling intervals, the reference FWHM (i.e., as measured in the motion-free reconstruction) was reached in less than eight iterations using kernel *K*_5_. As an alternative to the RMD, the residual motion kernel could also be directly incorporated in the iterative reconstruction by including it as an image space resolution model in the system matrix [20].

We found that using pose estimates interpolated from the tracking device pose measurements at a time resolution of 0.2 ms (list-mode data time resolution) show similar spatial resolution improvement to the RMD. Similarly, Zhou et al. [27] found that pose interpolation slightly increased the contrast in the motion corrected reconstructions. The difference of the resolution phantom rods FWHM using RMD and interpolated poses was less than 0.03 mm. The implementation of RMD requires greater effort than interpolation of the pose measurements. However, RMD can give additional information that can serve to define the most appropriate motion tracking sampling interval.

The RMD can be of particular relevance for animal studies where animals in a particular condition group show increased motion speed compared to the other condition groups, e.g., animals with motor disabilities or with abnormal motion behavior as in the case of epilepsy models. This scenario was shown in the numerical mouse brain phantom simulation.

While the RMD results in improved resolution, there is still some loss of resolution compared to the motion-free reconstruction. In our experiments, the resolution loss was higher in the phantom experiments than in the simulation experiments. This can be attributed to factors degrading the spatial resolution that were not simulated in the simulation experiments such as the tracking error, spatial calibration error, and the time synchronization delay. In the case of higher motion speed, in addition to the greater finite motion sampling error, higher tracking error can arise due to an increase in the camera image blurring. It is conceivable that also for these factors one could calculate a deconvolution kernel in a similar fashion as we have calculated the residual motion kernel.

Finally, the residual motion kernel can serve to assess the loss of spatial resolution in a single voxel, e.g., at the center of the region of interest. In cases where the motion sampling interval can be adjusted, the optimal tracking frame rate can be selected by choosing the motion sampling interval for which the deconvolution kernel principal axis magnitude is low in the central voxel of the region of interest. It should be noted that a trade-off has to be made. Indeed, increasing the motion sampling rate will reduce the resolution loss due to finite motion sampling in the noise free case. However, in practice, the short time frames will also degrade the quality of the motion estimates in individual frames due to noise. In our experiments, loss of spatial resolution due to finite motion sampling was observed for deconvolution kernels with principal axis average magnitude of about 0.4 mm (phantom 32 ms simulation experiment). Therefore, to avoid the effect of the finite motion sampling on the spatial resolution, it is recommended to reduce the sampling interval until the kernel principal axis magnitude is lower than 0.4 mm.

## Conclusions

The loss of spatial resolution in the motion corrected reconstructions caused by the residual motion depends on the subject motion and the tracking frame rate and is thus study dependent. This unwanted effect was demonstrated and was addressed by the residual motion deconvolution algorithm. This RMD algorithm uses a study dependent and spatially variant residual motion deconvolution kernel to reduce the motion dependent resolution loss caused by the residual motion. In resolution phantom experiments, the RMD method improved the motion corrected reconstructions spatial resolution. For awake unconstrained animal studies where the motion can be considerably different between subjects, the proposed method can obtain results that are less affected by the particular type of motion of the study or the tracking frame rate.
